# Effect of budesonide/glycopyrronium/formoterol fumarate dihydrate on cardiopulmonary outcomes in COPD: rationale and design of the THARROS trial

**DOI:** 10.1183/23120541.00324-2025

**Published:** 2025-12-22

**Authors:** Fernando J. Martinez, John R. Hurst, MeiLan K. Han, David Price, Jinping Zheng, David D. Berg, Michel Pszczol, Mona Bafadhel, Carolyn S.P. Lam, Martin Fredriksson, Martin R. Cowie, Niki Arya, Karin Bowen, Alec Mushunje, Mehul Patel

**Affiliations:** 1 Division of Pulmonary, Allergy, and Critical Care Medicine, University of Massachusetts Chan, Worcester, MA, USA; 2 UCL Respiratory, University College London, London, UK; 3 Division of Pulmonary and Critical Care, University of Michigan, Ann Arbor, MI, USA; 4 Observational and Pragmatic Research Institute, Singapore, and Centre of Academic Primary Care, Division of Applied Health Sciences, University of Aberdeen, Aberdeen, UK; 5 State Key Laboratory of Respiratory Disease, National Clinical Research Centre for Respiratory Disease, Guangzhou Institute of Respiratory Health, First Affiliated Hospital of Guangzhou Medical University, Guangzhou, China; 6 Brigham and Women's Hospital, Harvard Medical School, Boston, MA, USA; 7 Patient Author, Rio de Janeiro, Brazil; 8 King's Centre for Lung Health, School of Immunology and Microbial Sciences, Faculty of Life Sciences and Medicine, King's College London, London, UK; 9 National Heart Centre Singapore, Duke-National University of Singapore, Singapore; 10 Clinical Development, Late Clinical Cardiovascular Renal and Metabolism, BioPharmaceuticals R&D, AstraZeneca, Gothenburg, Sweden; 11 Clinical Development, Late Cardiovascular Renal and Metabolism, BioPharmaceuticals R&D, AstraZeneca, Boston, MA, USA; 12 Biometrics, Respiratory and Immunology, BioPharmaceuticals R&D, AstraZeneca, Durham, NC, USA; 13 Biometrics, Respiratory and Immunology, BioPharmaceuticals R&D, AstraZeneca, Gaithersburg, MD, USA; 14 Clinical Development, Respiratory and Immunology, BioPharmaceuticals R&D, AstraZeneca, Cambridge, UK

## Abstract

**Background:**

COPD and cardiovascular disease (CVD) are leading causes of death with overlapping and syndemic pathophysiological interactions. Inhaled triple therapies containing inhaled corticosteroids (ICS), long-acting muscarinic antagonists (LAMA) and long-acting β_2_-agonists (LABA) reduce COPD exacerbation rates and improve lung function *versus* dual LAMA/LABA therapy. The effect of inhaled triple therapies on combined cardiac and pulmonary (*i.e.*, cardiopulmonary) events in people with COPD and elevated cardiopulmonary risk has not been prospectively tested in randomised clinical trials.

**Methods:**

THARROS is a multinational, event-driven cardiopulmonary outcomes trial evaluating budesonide/glycopyrronium/formoterol fumarate dihydrate (BGF) triple therapy *versus* glycopyrronium/formoterol fumarate dihydrate dual therapy in patients with COPD and elevated cardiopulmonary risk not using ICS-containing maintenance therapy. Eligibility requirements include symptomatic COPD (COPD Assessment Test scores ≥10) without a requirement for prior COPD exacerbations, blood eosinophils ≥100 cells·mm^−3^, established CVD or CVD risk based on clinical characteristics, clinical risk scores or imaging-based risk criteria. The composite primary end-point is time to first severe cardiac or COPD event and includes three event types, including severe cardiac events (heart failure acute healthcare visit/hospitalisation, myocardial infarction hospitalisation), severe COPD exacerbations and cardiopulmonary death. Approximately 5000 patients will be randomised to achieve 632 participants with ≥1 primary severe adjudicated cardiopulmonary event.

**Conclusion:**

This first-of-its-kind cardiopulmonary outcomes trial will determine the effect of BGF on a novel composite end-point comprising severe cardiopulmonary events in a broad COPD population with elevated cardiopulmonary risk not currently using ICS-containing maintenance therapy.

## Shareable abstract

**THARROS, a first-of-its-kind event-driven cardiopulmonary trial, will assess BGF benefits *versus* dual therapy with GFF on cardiopulmonary outcomes in a COPD population with elevated cardiovascular risk and who are not on ICS-containing maintenance therapy.** https://bit.ly/4dK58Tw

## Graphical abstract

**Figure:**
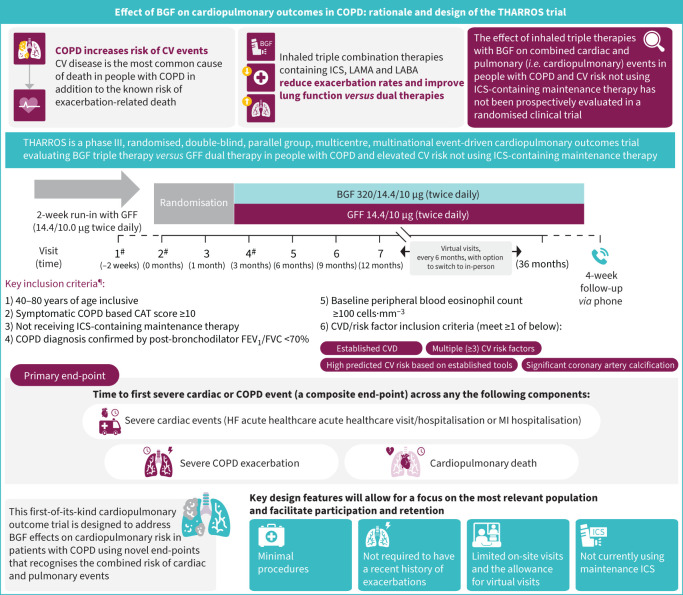
Effect of budesonide/glycopyrrolate/formoterol fumarate (BGF) on cardiopulmonary outcomes in COPD: rationale and design of the THARROS trial. CAT: COPD Assessment Test; CV: cardiovascular; CVD: cardiovascular disease; FEV_1_: forced expiratory volume in 1 s; FVC: forced vital capacity; GFF: glycopyrrolate/formoterol fumarate; HF: heart failure; ICS: inhaled corticosteroid; LABA: long-acting β_2_-agonist; LAMA: long-acting muscarinic antagonist; MI: myocardial infarction. ^#^: In-person visit; others may be virtual or in-person. Each person expected to be followed for up to 3 years (3-month minimum) or until a pre-defined number of events is achieved; the approximate population size will be 5000 depending on event and recruitment rate. ^¶^: Among those included for participation, ≥50% must have established CV disease.

## Introduction

COPD and cardiovascular disease (CVD) are leading causes of death globally [1] and share overlapping pathophysiological drivers [2, 3], having a syndemic relationship [4]. People with COPD are at increased cardiovascular (CV) event risk [5–8], including myocardial infarction (MI) [5, 6, 8] and heart failure (HF) [6, 9], with risk particularly exaggerated surrounding exacerbations [7, 8, 10].

Compared with respiratory-related deaths, CV-related deaths are more prevalent in people with COPD having Global Initiative for Chronic Obstructive Lung Disease (GOLD) spirometric grades indicating mild/moderate disease (group 1 (forced expiratory volume in 1 s (FEV_1_) ≥80% predicted); group 2 (50% predicted≤FEV_1_ <80% predicted)), while respiratory deaths are more prevalent in patients with GOLD classification severe/very severe disease (group 3 (30% predicted≤FEV_1_ <50% predicted); group 4 (<30% predicted)) [11, 12]. Furthermore, all-cause mortality has been shown to be increased in people with COPD who have features of impaired left ventricular filling or heart failure with preserved ejection fraction [13]. This emphasises the importance of evaluating and managing CV risk in COPD. Although CVD is a common cause of death in people with COPD [14–16], opportunity analyses from the CONQUEST study show only one in five patients without a history of cardiac disease had a cardiac risk assessment [17]. The relevance of cardiac risk in COPD is contextualised by the fact that exacerbation prevention may reduce the risk of cardiopulmonary events (*i.e.*, a combination of cardiac and/or pulmonary events such as MI, HF and exacerbations), thereby reducing mortality in people with COPD [18, 19].

Inhaled triple combination therapies containing an inhaled corticosteroid (ICS), long-acting muscarinic antagonist (LAMA) and long-acting β_2_-agonist (LABA) reduce COPD exacerbation rates and improve lung function *versus* dual therapies [20–24]. In ETHOS, a study of patients with moderate-to-very severe COPD and an exacerbation history in the previous year, fixed-dose triple therapy with budesonide/glycopyrronium/formoterol fumarate dihydrate (BGF) 320.0/14.4/10.0 µg (equivalent to budesonide/glycopyrrolate/formoterol fumarate 320.0/18.0/9.6 µg) reduced moderate/severe exacerbations [20] and improved lung function [21] *versus* LAMA/LABA therapy with glycopyrronium/formoterol fumarate dihydrate (GFF) 14.4/10.0 µg and ICS/LABA therapy with budesonide/formoterol fumarate dihydrate (BFF) 320/10 µg. Similarly, in KRONOS, a study of patients with moderate-to-very severe COPD and no exacerbation history requirement, BGF 320.0/14.4/10.0 µg improved lung function *versus* GFF, BFF and open-label budesonide/formoterol fumarate dihydrate *via* dry-powder inhaler and reduced moderate/severe exacerbation rates *versus* GFF [22]. In IMPACT, a study of patients with COPD and an exacerbation history in the previous year, triple therapy with fluticasone furoate (FF)/umeclidinium (UMEC)/vilanterol (VI) resulted in lower moderate/severe exacerbation rates *versus* dual FF/VI or UMEC/VI [23]. ETHOS, KRONOS and IMPACT allowed inclusion of participants on ICS before study entry [21–23].

In ETHOS, although not statistically significant due to placement in the testing hierarchy, the regulatory-agency approved BGF dose (320.0/14.4/10.0 µg) was associated with lower all-cause mortality risk *versus* dual LAMA/LABA therapy with GFF (14.4/10.0 µg) [14, 20]. Importantly, even though people with severe cardiac disease (*e.g.*, highly symptomatic HF, recent MI, significantly abnormal electrocardiogram, clinically uncontrolled hypertension) were excluded from ETHOS, two notable CV-related findings were observed. First, CV-related deaths were the most common cause of death in this population of individuals with COPD [14], emphasising the importance of managing CVD in COPD. Second, major adverse cardiac events were numerically lower with BGF *versus* GFF [14, 25], supporting the potential benefit of BGF over GFF on CV-related outcomes. *Post hoc* analyses from the EUROSCOP study support the concept that ICS can provide cardiac benefits in people with COPD, as fewer people with mild COPD treated with budesonide experienced ischaemic cardiac events *versus* those receiving placebo [26]. However, there is currently no designated randomised controlled trial (RCT) evidence for inhaled medications reducing CV events in people with COPD. In the SUMMIT trial, a prospective RCT that recruited a moderate COPD population with CV risk and evaluated treatment with FF, VI and FF/VI against placebo, FF and FF/VI did not affect mortality or CV outcomes [27].

Given the high frequency of combined severe cardiac or pulmonary events (cardiopulmonary events), there remains an unmet need to explicitly target reducing cardiopulmonary events [18] and disease-related mortality in people with COPD. In the THARROS outcome study, which is an event-driven study where estimated sample size and study duration is dependent on the occurrence of a sufficient number of events for the primary end-point, we will test the hypothesis that BGF triple therapy *versus* GFF dual therapy decreases cardiopulmonary events in people with COPD and elevated cardiopulmonary risk who are not currently using ICS maintenance therapy. GFF was chosen as the study comparator as it is the expected standard of care in those with ongoing COPD symptoms [28]. Given that cardiopulmonary outcomes are fundamental features and intricately related in people with COPD, the novel cardiopulmonary outcome measure in THARROS will assess key clinical deteriorations that capture cardiac-related and COPD-related hospitalisation and mortality in people with symptomatic COPD and elevated cardiopulmonary risk. Key design features (*i.e.*, inclusion of only those not currently using maintenance ICS, not restricting entry to those with exacerbation histories, limiting on-site visits and allowing for virtual visits and minimal procedures) will help focus on the most relevant population and facilitate participation and retention. The data obtained from THARROS may have a significant impact on clinical practice as it relates to COPD management (see the supplemental material for plain language summary infographic).

## Methods

### Study design

The THARROS protocol conforms with items in the Standard Protocol Items: Recommendations for Interventional Trials (SPIRIT) checklist and the World Health Organization Trial Registration Data Set (see supplemental tables 1 and 2), with the description below aligning with SPIRIT items 8–33.

THARROS (NCT06283966) is a phase III, randomised, double-blind, parallel group, multicentre, multinational event-driven cardiopulmonary outcome trial including people with COPD and elevated cardiopulmonary risk not currently using ICS-containing maintenance therapy. The study will be conducted in a planned 37 countries (see supplemental table 2 for a full list). All participants will be required to provide informed consent to participate. After visit 1, study medication will be shipped directly to the participant's home or the study site (per local regulations and requirements, and per investigator and/or participant preference) following the randomisation visit (visit 2).

**FIGURE 1:**
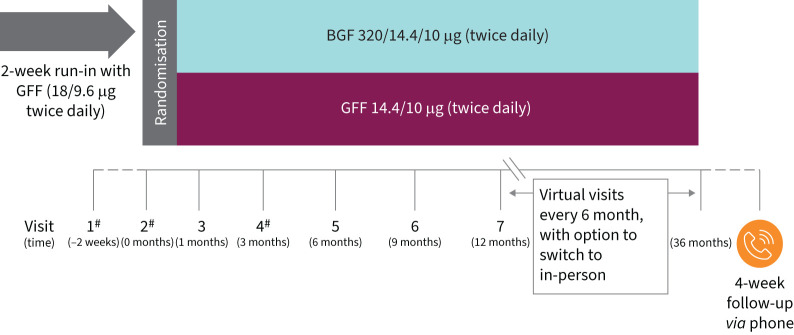
THARROS study design. BGF: budesonide/glycopyrrolate/formoterol fumarate; GFF: glycopyrrolate/formoterol fumarate. ^#^: In-person visit; others may be virtual or in-person. Each person expected to be followed for up to 3 years (minimum of 3 months) or until a pre-defined number of events is achieved; the approximate population size will be 5000 depending on event and recruitment rate.

The study consists of three periods, as follows: a 2-week screening period with a GFF 14.4/10.0 μg run-in, a double-blind treatment period with participants randomised to BGF *via* metered-dose inhaler (MDI) 320.0/14.4/10.0 μg (equivalent to budesonide/glycopyrrolate/formoterol fumarate 320/18/9.6 µg) or GFF *via* MDI 14.4/10 μg for up to 3 years (3-month minimum) and a 4-week follow-up period for a total study duration of up to 37 months (figure 1). During the run-in phase, participants will initiate GFF 14.4/10.0 μg twice daily until the evening before visit 2 (*i.e.*, randomisation). At visit 1, participants will receive salbutamol for rescue use during the study. Adherence will be assessed using dose counters at site and virtual visits in addition to direct questioning at virtual visits and will be appropriately documented.

Double-blind treatment begins at visit 2 (figure 1), when eligible participants will be randomised 1:1 to BGF or GFF for an estimated duration of up to 3 years and a minimum 3-month duration. Participants will be randomised using a centralised Interactive Response Technology (IRT)/Randomization and Trial Supply Management (RTSM) system, with sites providing telephone contacts and/or system login information. Following randomisation, participants will undergo up to nine additional visits, either at the study site or virtually. Visits 1 (screening), 2 (randomisation) and 4 will be conducted in-person at the study site. All other visits may be completed virtually, with the option to switch any virtual visit to an in-person visit. The virtual visit option is a key study feature aligned to the input of potential participants, who indicated virtual visits are convenient and utilisation of personal devices would not be a major barrier.

The study is event-driven with each participant expected to be followed for up to 3 years for efficacy and safety assessments, or until 632 participants have ≥1 primary severe adjudicated cardiopulmonary event that informs the primary end-point, if earlier. When the required event number is reached, all randomised participants will be scheduled for a study closeout visit (SCV) to assess primary and secondary objectives in those randomised within 3 years of the SCV. Vital status will be assessed once it is predicted that 632 participants have had ≥1 severe adjudicated event contributing to the primary end-point. All randomised participants will be followed from randomisation until study completion, SCV or death (whichever comes first), regardless of discontinuation of study intervention, change in respiratory or CV therapy or the occurrence of a non-fatal severe cardiopulmonary event in the composite end-point. The study will be quadruple-blinded (patients, care provider, investigator, outcome assessor), with the IRT/RTSM programmed with blind-breaking instructions.

Post-treatment safety follow-up will occur 4 weeks after the last study medication dose in participants who complete or withdraw from the study, discontinue study intervention or who are still on study intervention at the SCV. Participants may withdraw at any time for any reason and will be considered lost to follow-up if they repeatedly fail to return for scheduled visits and are unable to be contacted. If a participant withdraws consent for follow-up, investigators will make efforts to determine the participant's health status, including vital status and time of death (if applicable), where permitted by local regulations.

This study will be conducted in accordance with the consensus ethical principles derived from the Declaration of Helsinki (as amended at 64th WMA General Assembly, Fortaleza, Brazil, October 2013), Council for International Organisations of Medical Sciences International Ethical Guidelines, all applicable International Council for Harmonisation (ICH) Good Clinical Practice (GCP) guidelines and applicable laws and regulations. All relevant documents will be submitted to an institutional review board (IRB) or independent ethics committee (IEC) and approved before study initiation. Before enrolment, participants or their legally authorised representatives will be required to sign an informed consent statement that meets regulations for privacy and data collection. Per GCP requirements, protocol amendments will be submitted for relevant regulatory and IRB/IEC review before deployment to sites, with relevant informed consent form amends to ensure participants are updated and provide consent.

### Participants

Tables 1 and 2 summarise inclusion and exclusion criteria. The study will include participants aged 40‒80 years with symptomatic COPD (based on COPD Assessment Test (CAT) score) and with cough and sputum CAT sub-scores ≥2, as productive cough is associated with increased cardiopulmonary and exacerbation risk [29, 30], who are current or former smokers (history of ≥10 pack-years of cigarette smoking) and who have not used maintenance ICS in the prior 12 months and are at risk of a cardiopulmonary event (see end-points below). For THARROS, a COPD diagnosis will be confirmed by a post-bronchodilator FEV_1_/forced vital capacity <0.70. This study will include a primary prevention approach in which eligible participants will not be required to have experienced previous cardiac or COPD events. Participants with an active asthma diagnosis within the past 5 years (previous child or adolescent diagnoses allowed) and asthma–COPD overlap will be excluded. Prohibited medications requiring cessation before screening will include (but not be limited to) ICS-containing maintenance therapy (12 months), systemic anticholinergics (7 days), corticosteroids (maintenance/prophylactic oral or *intravenous*: 8 weeks; injectable systemic: 3 months) and other investigational drugs (30 days or five half-lives, whichever longer).

**TABLE 1:** THARROS inclusion criteria

Category	Criteria
**Age-related**	Male or female participants 40–80 years of age (inclusive)
**Respiratory-related**	•Symptomatic COPD based on CAT score ≥10, with cough and sputum component sub-scores ≥2 •Not receiving ICS-containing maintenance therapy in the prior 12 months •Post-bronchodilator FEV_1_/FVC <70% predicted •Current or former smokers (history of ≥10 pack-years of cigarette smoking) •Baseline peripheral blood eosinophil count ≥100 cells·mm^−3^
**Cardiovascular-related**	Must fulfil at least one of the following four criteria:
	**1)** Established CVD (meet ≥1 of below; at least 50% of participants must have established CVD, as reflected by clinical characteristics, clinical risk scores or imaging-based risk criteria): •Angina pectoris with evidence of myocardial ischaemia •Myocardial infarction •Percutaneous coronary intervention; coronary artery bypass grafting •Objective findings of coronary stenosis (≥50%) in at least two coronary artery territories involving the main vessel, a major branch or a bypass graft •Chronic HF with NYHA class II–III functional limitation at visit 1^#^ •Peripheral arterial disease (any of the following): peripheral arterial intervention, stenting surgical revascularisation; lower extremity amputation as a result of peripheral arterial obstructive disease; current symptoms of intermittent claudication and ankle/branchial index (<90 documented within last 12 months); and angiographic evidence of peripheral artery disease
	**2)** Multiple CV risk factors (meet ≥3 of the following): •Hypertension (at least one of the following): documented history (previous 6 months) of BP >140/90 mmHg confirmed at visit 1 with both elevated systolic (>140 mmHg) and diastolic (>90 mmHg) BP on the last two of three measurements or receiving ≥1 antihypertensive therapy prescribed by a physician for BP lowering •History of diabetes mellitus •History of chronic kidney disease, with eGFR ≥20 mL·min^−1^·1.73 m^−2^ and <60 20 mL·min^−1^·1.73 m^−2^ confirmed at visit 1 •History of dyslipidaemia (previous 12 months) with at least one of the following: LDL-C >130 mg·dL^−1^ (3.36 mmol·L^−1^) at visit 1; HDL-C <40 mg·dL^−1^ (1.03 mmol·L^−1^) for men or <50 mg·dL^−1^ , (1.29 mmol·L^−1^) for women at visit 1; and on physician-prescribed lipid lowering therapy for hypercholesterolaemia (LDL-C >130 mg·dL^−1^ (3.36 mmol·L^−1^)) for 12 months •History of obesity with confirmation of BMI ≥30 kg·m^−2^ at visit 1
	**3)** High predicted CV risk based on established risk tools for participants without established CVD within 6 months of visit 1:^¶^ •Q-Risk-3 score >20%, •ASCVD risk equation score >20% •Framingham risk score >20% •SCORE2 tool >7.5% for <50 years of age, >10% for 50 to 69 years of age, SCORE2-OP tool >15% for >70 years of age
	**4)** Documented coronary artery calcification: •Based on visual assessment by a radiologist or other appropriately qualified individual and/or quantitative scoring (*e.g.*, Agatston scoring (moderate ≥101–≤1000, severe/heavy >1000 [33]) where available •At least a moderate coronary artery calcification score plus any one other CV criteria listed above •A severe/heavy coronary artery calcification score
**Other**	•Willing and able to adjust current COPD therapy •Demonstrate acceptable MDI administration •Willing to visit at the study site or participate in virtual visits •Female is of nonchildbearing potential (either permanently sterilised or is post-menopausal), childbearing potential (has a negative serum pregnancy test at visit 1 and must use one highly effective form of birth control)

ASCVD: atherosclerotic cardiovascular disease; BMI: body mass index; BP: blood pressure; CAT: COPD Assessment Test; CV: cardiovascular; CVD: cardiovascular disease; ICS: inhaled corticosteroid; eGFR: estimated glomerular filtration rate; FEV_1_: forced expiratory volume in 1 s; FVC: forced vital capacity; HF: heart failure; HDL-C: high-density lipoprotein cholesterol; LDL-C: low-density lipoprotein cholesterol; MDI: metered-dose inhaler; NYHA: New York Heart Association; SCORE2: Systematic Coronary Risk Evaluation; SCORE2-OP: Systematic Coronary Risk Evaluation-Older Persons. ^#^: No more than 10% of participants should enter through only the HF criteria; NYHA class II is defined as participants comfortable at rest; ordinary physical activity results in fatigue, palpitations, breathlessness or angina pectoris and class III is defined as participants comfortable at rest, but with less than ordinary physical activity causing fatigue, dyspnoea, palpitations or angina. ^¶^: CV risk tools utilised in other countries/regions indicating equivalent risk (high on the scale) will also be accepted.

**TABLE 2:** THARROS exclusion criteria

Category	Criteria
**Respiratory**-**related**	Active diagnosis of asthma within the past 5 years (previous diagnosis as a child or adolescent are eligible), asthma–COPD overlap Any other chronic respiratory disease other than COPD, such as alpha-1 antitrypsin deficiency, active tuberculosis, lung fibrosis, sarcoidosis, interstitial lung disease and pulmonary hypertension History of lung transplant or actively listed for transplant Pneumonia and/or moderate or severe COPD exacerbation 8 weeks prior to visit 1^#^ Use of maintenance ICS treatment within the past 12 months Participants with known hypersensitivity to LAMA, LABA or ICS or any component of MDI
**Cardiovascular-related**	History of heart transplant or actively listed for transplant Implanted left ventricular assist device or implant anticipated in <3 months Unstable or life-threatening cardiac disease, including an MI or unstable angina in last 8 weeks and unstable or life-threatening cardiac arrhythmia requiring intervention in past 8 weeks^#^
**Other**	End-stage renal disease requiring renal replacement therapy or eGFR <20 mL·min^−1^·1.73 m^−2^ History of lung cancer and/or treatment for lung cancer within the 5 years prior to visit 1 Any life-threatening condition with a life expectancy <5 years Unable to abstain from protocol-defined prohibited medications Participation in another clinical study Currently pregnant or breastfeeding (females)

eGFR: estimated glomerular filtration rate; ICS: inhaled corticosteroid; LABA: long-acting β_2_-agonist; LAMA: long-acting muscarinic antagonist; MDI: metered-dose inhaler; MI: myocardial infarction. ^#^: Any participant who experiences unstable or life-threatening cardiac disease, pneumonia and/or moderate or severe COPD exacerbation during the run-in period will be excluded, but can be rescreened 8 weeks after the event's resolution.

Other respiratory-related criteria include only allowing those without a history of maintenance ICS use in the previous 12 months to participate (to more directly evaluate the impact of ICS step-up on cardiopulmonary events), not requiring an exacerbation history and including only those with blood eosinophils >100 cells·mm^−3^ to align with clinical considerations [28, 31] of those likely to benefit from triple ICS-containing therapies [20, 22, 32].

The trial will include those needing primary CV prevention (*i.e.*, with CV risk factors in the absence of established CVD) and secondary CV prevention (*i.e.*, in the setting of established CVD). As detailed in table 1, participants must meet ≥1 of 4 CV disease/risk factor criteria, as follows: 1) established CVD, 2) ≥3 CV risk factors, 3) high predicted CV risk based on established risk tools for those without established CVD, or 4) documented coronary artery calcification based on visual assessment by a radiologist or appropriately qualified individual and/or quantitative scoring (*e.g.*, Agatston scoring (moderate: ≥101–≤1000; severe/heavy: >1000 [33]). Overall, ≥50% of participants must have established CVD, with an additional restriction on the proportion of participants with heart failure to ensure CV component balance. Recruitment in any of the CV risk categories may be closed if that risk category is not contributing sufficiently to observed blinded event rates.

### End-points

Table 3 summarises the key primary and secondary end-points for THARROS. The primary end-point will consist of a novel composite end-point that captures severe cardiac and pulmonary effects of COPD. The study primary composite end-point is time to first severe cardiac or COPD event end-point across any of the following three groups of events: severe cardiac events (including HF acute healthcare visit/hospitalisation, MI hospitalisation), severe COPD exacerbation (*i.e.*, requiring hospitalisation) or cardiopulmonary death. The cardiac-related component of cardiopulmonary death is defined by modified criteria from Hicks *et al*. [34] and includes sudden cardiac death and death from acute MI (including MI treatment procedures) or from HF. The pulmonary-related component of cardiopulmonary death includes death from COPD with or without pneumonia, pulmonary embolism, pneumothorax, pulmonary haemorrhage, pneumonia without COPD exacerbation and respiratory failure. All potential primary analysis events will be adjudicated by an independent Clinical Endpoint Committee (CEC) comprising pulmonologists, cardiologists and other medical specialties (as needed). Secondary end-points will assess a range of cardiac and pulmonary outcomes including time to first severe COPD exacerbation event, severe COPD exacerbation rate, time to first severe cardiac event, severe cardiac event rate, time to cardiopulmonary death, moderate/severe COPD exacerbation rate, time to first moderate or severe COPD exacerbation event, time to MI hospitalisation or cardiac death and time to HF hospitalisation/urgent care or cardiac death.

**TABLE 3:** THARROS primary estimands and end-points

Estimand component	Description
**Treatment**	Randomised treatment of BGF twice daily or GFF twice daily
**Population**	Adults (40–80 years of age) with COPD not receiving ICS and with elevated cardiopulmonary risk
**Primary end-point**	Time to first severe cardiac or COPD event (a composite end-point) across any of the following components: •Severe cardiac events (HF acute healthcare visit/hospitalisation or MI hospitalisation) •Severe COPD exacerbation •Cardiopulmonary death^#^
**Population-level summary**	Hazard ratio (BGF MDI 320.0/14.4/10.0 μg *versus* GFF MDI 14.4/10.0 μg)
**Handling of intercurrent events**	•Treatment discontinuation: treatment policy •Step-up to triple therapy: treatment policy •Change in pulmonary or CV therapy: treatment policy •Death due to noncardiopulmonary cause in absence of other components: participant will be censored at the time of death

BGF: budesonide/glycopyrronium/formoterol fumarate dihydrate; CV: cardiovascular; GFF: glycopyrronium/formoterol fumarate dihydrate; HF: heart failure; ICS: inhaled corticosteroid; MDI: metered dose inhaler; MI: myocardial infarction. ^#^: Cardiac-related death includes sudden cardiac death and death due to acute MI (including procedures to treat MI) or HF; pulmonary-related deaths include COPD with or without pneumonia, pulmonary embolism, pneumothorax, pulmonary haemorrhage, pneumonia in absence of COPD exacerbation, respiratory failure and those defined by the Clinical Endpoint Committee; noncardiac/nonpulmonary death defined as any death with a specific cause that is not adjudicated to be cardiac or pulmonary in nature.

An independent data monitoring committee (DMC) will monitor the benefit/risk throughout the study and will operate in accordance with a DMC charter.

#### Safety

This study will assess safety and tolerability end-points, including adverse events (AEs) leading to study drug discontinuation, serious AEs, AEs of special interest (AESIs) that include pneumonia-related hospitalisation or death and laboratory assessments. The CEC will adjudicate AESIs of pneumonia-related hospitalisation or death. The sponsor is responsible for participant safety, including providing support for any unexpected events during the study.

### Statistical analysis

Approximately 8334 participants will be screened to achieve the approximately 5000 randomised participants expected to provide the required number of primary events, but the final screened and randomised sample size will ultimately depend on observed screen failures and primary end-point event rates. This population differs significantly from those studied in previous triple therapy clinical trials, such as ETHOS and IMPACT (where severe cardiac disease was generally excluded, a recent COPD exacerbation history was required and those on ICS maintenance therapy were included). Subsequently, cardiac and pulmonary event rates will be evaluated across a broad COPD population not on maintenance ICS therapy. Prior randomised controlled trials and modelling using the Optimum Patient Care Research Database (https://opcrd.optimumpatientcare.org) informed the expected incidence rate estimates.

Targeting a power of 80%, a two-sided significance level of 4.97% (conditional on one interim analysis) and assuming a true hazard ratio (HR) of 0.8 for the BGF *versus* GFF comparison that accounts for study intervention discontinuation, 632 events are needed to demonstrate statistical significance for the comparison of time to first severe cardiac or COPD event. A sample size of approximately 5000 (2500 per treatment arm) is expected to provide the target number of events assuming a 7.5% annual incidence in the comparator arm.

HR point estimates, associated 95% confidence intervals and p-values will be provided for time to event comparisons. Rate ratio point estimates, associated 95% confidence intervals and p-values will be provided for recurrent event comparisons. A multiple testing procedure will be used for hierarchical testing of the primary and secondary end-points (see the supplemental material for details).

For the primary end-point (time to first severe cardiac or COPD event), a Cox proportional hazards model will be used, with an HR and corresponding 95% confidence intervals presented. The model will include region and the log of baseline eosinophils as covariates. All data (up until 36 months from randomisation) will be analysed according to randomised treatment regardless of treatment discontinuation or subsequent COPD or CV therapy. All events in the primary analysis will be adjudicated as described above and graphically summarised using Kaplan–Meier plots. The supplemental material provides additional description of secondary and supportive analyses.

### Data management and auditing

All data will be entered electronically at the site where the data originates, kept on file at the site and maintained in storage for 25 years after study completion. Modifications to data written to the database will be documented through either the data change system or an inquiry system. Written documentation of changes will be available *via* electronic logs and audit trails. The electronic case report form has audit trails for all data entered by the site or queried by study team. Audit checks (by an independent internal quality check department and by external GCP audits) will be implemented so planned and systematic actions established to ensure the trial is performed and the data are generated, documented/recorded, and reported comply with GCP and regulatory requirements*.* The primary investigators will review the full clinical study report with access to all available data.

## Discussion

THARROS is a first-of-its-kind cardiopulmonary outcome trial designed to assess BGF benefit *versus* GFF in people with COPD using novel end-points that recognise combined severe cardiac and pulmonary event risk in people with COPD not receiving maintenance ICS and with elevated cardiopulmonary risk. THARROS will address important questions about the benefits of ICS/LAMA/LABA with BGF *versus* LAMA/LABA with GFF on cardiopulmonary outcomes. As there are limited data on treatment differences between ICS/LAMA/LABA and LAMA/LABA therapies among those with no recent ICS use, THARROS will also provide important information on the benefits of BGF triple therapy in this population of people with COPD.

The relevance of assessing BGF treatment effects on cardiopulmonary outcomes in people with COPD is supported by a recent *post hoc* analysis of the ETHOS study [35]. In that *post hoc* analysis, BGF reduced the rate of first occurrence of CV and severe cardiopulmonary events *versus* GFF, including for CV-related AESIs, cardiac AEs and severe cardiopulmonary events that comprised major adverse cardiac events, severe exacerbations and nonmalignant respiratory-related death. THARROS will extend these findings by prospectively focusing on those with elevated cardiopulmonary risk not currently using maintenance ICS.

There are multiple study design features contributing to the novelty of THARROS. Two such features are including participants without requiring an exacerbation history and requiring participants to not be receiving maintenance ICS therapy, as the individuals are less likely to step-up to triple therapy per GOLD 2024 recommendations [28]. This is a strength because targeting individuals not currently receiving maintenance ICS will more definitively evaluate the efficacy of ICS-containing triple therapy on cardiopulmonary events. Additionally, it allows for a focus on end-points beyond lung function and exacerbations, which have been the focus of previous ICS/LAMA/LABA triple therapy COPD studies [20–22]. To date, KRONOS is the only large clinical study that assessed BGF *versus* dual therapy without requiring an exacerbation history and showed treatment benefits for BGF *versus* GFF on exacerbations and lung function [22]. Based on patient input into the THARROS design, the study will also employ a combination of on-site and virtual visits that will increase the convenience of participation.

THARROS will also include individuals on LAMA or LABA monotherapy, as dual maintenance therapy is not an inclusion criterion. Hence, THARROS will provide data in this less studied group, who per GOLD 2024 recommendations [28] would not be escalated to ICS-containing triple therapy unless blood eosinophil levels are ≥300 cells·mm^−3^ or repeated exacerbations are experienced. This allows for an assessment of cardiopulmonary events in participants who may never have had a severe CV or respiratory event. The inclusion of participants with blood eosinophils >100 cells·mm^−3^ also aligns with GOLD 2024 recommendations on those more likely to benefit from ICS-containing triple therapies [28].

The CV-related inclusion criteria account for an array of established CVD and CV-related risk factors, including cardiac risk scoring with validated assessment tools (*e.g.*, Q-Risk, Framingham risk) in those without clinically established CVD and entry through clinically significant coronary calcification. The requirement that ≥50% of participants with established CVD be included ensures sufficient enrichment for those at risk of CV events. Evaluating a combined cardiopulmonary outcome assessing key clinical deteriorations that captures an array of CV-related and pulmonary-related outcomes, including mortality, is important in people with COPD and CV risk given cardiopulmonary outcomes are fundamental features of COPD. Such risk is evidenced by studies demonstrating that people living with COPD are at increased risk of CV events, including for MI [5, 6, 8] and HF [6, 9], a risk exaggerated at the time of an exacerbation.

CV-related death is a major cause of mortality among those with COPD, in addition to the known risk of respiratory-related death [6, 12, 36, 37], emphasising the importance of evaluating and managing CV risk in COPD to mitigate mortality. As acknowledged by the GOLD 2024 report, the triple ICS-containing therapies of BGF in ETHOS [14] and FF/UMEC/VI in IMPACT [23] reduce mortality rates *versus* dual LAMA/LABA therapy. This could be related to the anti-inflammatory properties of ICS and the ability of long-acting bronchodilators to reduce hyperinflation [18, 19], two key mechanisms that link COPD and CVD that are exaggerated during COPD exacerbations [18, 19]. THARROS will further evaluate the broader benefit of budesonide, in the form of BGF, when added to dual bronchodilators in a novel population that are at risk of cardiopulmonary events.

## Conclusions

COPD and CVD have a severe impact on quality of life and ultimately can be associated with poor clinical outcomes, including hospitalisation and mortality. The event-driven THARROS study will assess the benefit of BGF triple therapy *versus* LAMA/LABA therapy with GFF on combined cardiopulmonary events in people with COPD and elevated cardiopulmonary risk not using maintenance ICS therapy. The evaluation of cardiopulmonary outcomes assessing key clinical deteriorations, including mortality, is important because they are fundamental features of COPD. As it is critically important to treat both the pulmonary and cardiac aspects of COPD, the novel design of THARROS will provide important data that are relevant to potential changes in clinical practice and will do so using a design that facilitates patient participation.

## Data Availability

Upon study completion, the results of the THARROS study will be presented at scientific conferences and/or published in peer-reviewed scientific publications. Data underlying the findings described in this manuscript may be obtained in accordance with AstraZeneca's data sharing policy described at: https://astrazenecagrouptrials.pharmacm.com/ST/Submission/Disclosure. Data for studies directly listed on Vivli can be requested through Vivli at www.vivli.org. Data for studies not listed on Vivli could be requested through Vivli at https://vivli.org/members/enquiries-about-studies-not-listed-on-the-vivli-platform/. The AstraZeneca Vivli member page is also available outlining further details: https://vivli.org/ourmember/astrazeneca/.

## Supplementary material

**Please note:** supplementary material is not edited by the Editorial Office, and is uploaded as it has been supplied by the author. Supplementary material 00324-2025.SUPPLEMENT Infographic 00324-2025.SUPPLEMENT2
