# Supplementary material for: Effect of budesonide/glycopyrronium/formoterol fumarate dihydrate on cardiopulmonary outcomes in COPD: rationale and design of the THARROS trial
Source: ERJ Open Res. 2025 Dec 22;11(6):00324-2025. doi: 10.1183/23120541.00324-2025 (PMC12720155; doi:10.1183/23120541.00324-2025)
Supplement: Supplementary file 1 [file 00324-2025.SUPPLEMENT.pdf]

Supplementary information

Statistical analysis

The type I error will be strongly controlled (at 0.05) using a combination of sequential and Hochberg approaches in a multiple testing strategy. The primary endpoint of time to first severe cardiopulmonary event will be tested first, then the secondary endpoints of time to first severe COPD exacerbation and time to first severe cardiac event will be tested using a Hochberg strategy, followed by sequential testing of the secondary endpoints of moderate/severe exacerbation rate and time to cardiopulmonary death.

For the secondary endpoints with a time to event approach, an approach similar to the primary endpoint will be used. Analyses of the individual components of the composite primary endpoint (severe cardiac events [i.e., HF or MI visit/hospitalisation], severe COPD exacerbations, cardiopulmonary death) will be conducted to support the conclusion that each of the individual components contribute to the composite primary endpoint.

For secondary/supportive analyses of exacerbation rates (severe or moderate/severe), exacerbation rates will be compared between treatment arms using a negative binomial model, with the same covariates specified for the primary model along with number of moderate/severe exacerbations in the prior 12 months. The logarithm of the time at risk for exacerbation in the study will be used as an offset variable in the model to adjust for different follow-up times between participants. The estimated treatment effect (i.e., the rate ratio of BGF versus GFF) and corresponding 95% CI for the rate ratio will be presented. In addition, the exacerbation rate, as well as differences in treatment rates and the corresponding 95% CI within each treatment group will be presented.

An interim analysis may be planned following confirmation of adjudication of 60% of the planned total number of primary events. If conducted, the interim analysis will be performed by an independent data monitoring committee.

TABLE S1: Administrative information per SPIRIT guidelines

**Section/item**	**Item**	**Description**
Title	1	Effect of budesonide/glycopyrronium/formoterol fumarate dihydrate on cardiopulmonary outcomes in COPD: rationale and design of the THARROS trial
Trial registration: primary registry	2a	ClinicalTrials.gov: NCT06283966
Trial registration: World Health Organization (WHO) Trial Registration data	2b	Please see specific items related to WHO Trial Registration Data Set (version 1.3.1) in Table S2
Protocol version and date	3	D5989C00001 version 2.0 (28 January 2024)
Funding	4	AstraZeneca
Roles and responsibilities:	5a	• Fernando Martinez (Coordinating investigator; strategic approach)<br>• Alec Mushunje (Sponsor content approver; overseeing study conduct and data collection)

protocol contributors		• Karin Bowen (Sponsor content approver; statistical approach)<br>• Mehul Patel (Sponsor content approver; strategic approach and population characteristics)
Roles and responsibilities: contact information	5b	Trial sponsor: AstraZeneca<br>AstraZeneca AB, 151 85 Södertälje, Sweden.<br>AstraZeneca K.K., 3-1, Ofuka-cho, Kita-ku, Osaka 530-0011, Japan<br>Contact: Mehul Patel (mehul.patel1@astrazeneca.com)
Roles and responsibilities: study sponsor	5c	The study sponsor (AstraZeneca) was involved in the study design; collection, management, analysis, and interpretation of data, and writing of the protocol. The decision to publish this material was a joint decision among the authors, including those who are employees of the Sponsor.
Roles and responsibilities: centers and committees	5d	**Data monitoring committee (DMC):**<br>An independent DMC will be utilized to monitor the benefit/risk throughout the study. The committee will operate in accordance with a DMC charter. The DMC will have access to the individual treatment codes and will be able to merge these with the collected study data while the study is ongoing, as required.<br>**Clinical endpoint committee (CEC):**<br>An independent CEC will be composed of experts in the field of respiratory, cardiology, and other appropriate medical specialties as needed who are not involved in the study conduct. This committee will be independent of

		AstraZeneca and the investigators and will be implemented to review, assess, and adjudicate all deaths and events that potentially contribute to the primary composite endpoint and adjudicate adverse events of special interest of pneumonia leading to hospitalization or death.

TABLE S2: Items from the World Health Organization Trial Registration Data Set

**Data category**	**Information**
Primary Registry and Trial Identifying Number	ClinicalTrials.gov: NCT06283966
Date of Registration in Primary Registry	21 February 2024
Secondary Identifying Numbers	• D5989C00001<br>• IND 118313<br>• EU CT Registry: 2023-507407-59
Source(s) of Monetary or Material Support	AstraZeneca
Primary Sponsor	AstraZeneca
Secondary Sponsor(s)	None
Contact for Public Queries	AstraZeneca Clinical Study Information Center: phone (1-877-240-9479), e-mail (information.center@astrazeneca.com)

Contact for Scientific Queries	Coordinating Investigator: Fernando J. Martinez, MD, MS, Division of Pulmonary, Allergy, and Critical Care Medicine, University of Massachusetts Chan, Worcester, MA, USA<br>AstraZeneca Clinical Study Information Center: phone (1-877-240-9479), e-mail (information.center@astrazeneca.com)
Public Title	A Study Evaluating the Efficacy of Budesonide, Glycopyrronium and Formoterol Fumarate Metered Dosed Inhaler on Cardiopulmonary Outcomes in Chronic Obstructive Pulmonary Disease (THARROS)
Scientific Title	A Randomized, Double-blind, Parallel Group, Multi-center, Phase III Study to Assess the Efficacy of Budesonide, Glycopyrronium, and Formoterol Fumarate Metered Dose Inhaler Relative to Glycopyrronium and Formoterol Fumarate MDI on Cardiopulmonary Outcomes in Chronic Obstructive Pulmonary Disease (THARROS)
Countries of Recruitment	Argentina, Australia, Austria, Brazil, Bulgaria, Canada, Chile, China, Colombia, Czechia, Denmark, Finland, France, Germany, Greece, Hungary, India, Italy, Japan, Republic of Korea, Malaysia, Mexico, Norway, Peru, Philippines, Poland, Romania, Serbia, Slovakia, Spain, Sweden, Taiwan, Thailand, Turkey, Ukraine, United Kingdom, United States
Health Condition(s) or Problem(s) Studied.	Chronic Obstructive Pulmonary Disease

Intervention(s) and description	• Interventions: BGF 320/14.4/10 µg twice daily, GFF 14.4/10 µg twice daily<br>• Description: After a 2-week screening period with a GFF 14.4/10 µg run-in, participants will be randomised to double-blind treatment with BGF 320/14.4/10 µg via MDI or GFF via MDI 14.4/10 µg for up to 3 years (3-month minimum), and a 4-week follow-up period for a total study duration of up to 37 months.
Key Inclusion and Exclusion Criteria	<u>Key inclusion criteria:</u><br>• Male or female participants 40–80 years of age (inclusive)<br>• Symptomatic COPD based on CAT score ≥10, with cough and sputum component sub-scores ≥2<br>• Not receiving ICS-containing maintenance therapy in the prior 12 months<br>• Post-bronchodilator FEV_1/FVC <70% predicted<br>• Current or former smokers (history of ≥10 pack-years cigarette smoking)<br>• Baseline peripheral blood eosinophil count ≥100 cells/mm^3<br>• Must fulfil at least 1 of the 4 criteria below<br>○ *Established CVD* (meet ≥1 of below; at least 50% of participants must have established CVD, as reflected by clinical characteristics, clinical risk scores or imaging-based risk criteria):<br>▪ Angina pectoris with evidence of myocardial ischemia

	▪ MI<br>▪ Percutaneous coronary intervention; coronary artery bypass grafting<br>▪ Objective findings of coronary stenosis (≥50%) in at least 2 coronary artery territories involving the main vessel, a major branch or a bypass graft<br>▪ Chronic HF with NYHA Class II-III functional limitation at Visit 1[a]<br>▪ Peripheral arterial disease (any of the following):<br>– Peripheral arterial intervention, stenting surgical revascularisation<br>– Lower extremity amputation as a result of peripheral arterial obstructive disease<br>– Current symptoms of intermittent claudication and ankle/branchial index (<90 documented within last 12 months)<br>– Angiographic evidence of peripheral artery disease<br>○ *Multiple CV risk factors (meet ≥3 of below):*<br>▪ Hypertension (at least one of the following):<br>– Documented history (previous 6 months) of BP >140/90 mmHg confirmed at Visit 1 with both elevated systolic (>140 mmHg) and diastolic (>90 mmHg) BP on the last 2 of 3 measurements

	– Receiving ≥1 antihypertensive therapy prescribed by a physician for BP lowering<br>▪ History of diabetes mellitus<br>▪ History of chronic kidney disease, with eGFR ≥20 mL/min/1.73 m^2 and <60 mL/min/1.73 m^2 confirmed at Visit 1<br>▪ History of dyslipidaemia (previous 12 months) with at least one of the following:<br>– LDL-C >130 mg/dL (3.36 mmol/L) at Visit 1<br>– HDL-C <40 mg/dL (1.03 mmol/L) for men or <50 mg/dL (1.29 mmol/L) for women at Visit 1<br>– On physician-prescribed lipid lowering therapy for hypercholesterolemia (LDL-C >130 mg/dL [3.36 mmol/L]) for >12 months<br>▪ History of obesity with confirmation of BMI ≥30 kg/m^2 at Visit 1<br>○ *High predicted CV risk based on established risk tools for participants without established CVD within 6 months of Visit 1*[b]<br>▪ Q-RISK-3 score >20%<br>▪ ASCVD risk equation score >20%<br>▪ Framingham risk score >20%

	▪ SCORE2 tool >7.5% for <50 years of age, >10% for 50 to 69 years of age; SCORE2-OP tool >15% for >70 years of age<br>○ *Documented coronary artery calcification*<br>▪ Based on visual assessment by a radiologist or other appropriately-qualified individual and/or quantitative scoring (e.g., Agatston scoring [moderate, ≥101 to ≤1000; severe/heavy, >1000) where available<br>– At least a moderate coronary artery calcification score plus any one other CV criteria listed above<br>– A severe/heavy coronary artery calcification score<br>• Willing and able to adjust current COPD therapy<br>• Demonstrate acceptable MDI administration<br>• Willing to visit at the study site or participate in virtual visits<br>• Female is of non-childbearing potential (either permanently sterilised or is post-menopausal), childbearing potential (has a negative serum pregnancy test at Visit 1 and must use one highly effective form of birth control)<br><u>Key exclusion criteria:</u>

	• Active diagnosis of asthma within the past 5 years (previous diagnosis as a child or adolescent are eligible), asthma-COPD overlap<br>• Any other chronic respiratory disease other than COPD, such as alpha-1 antitrypsin deficiency, active tuberculosis, lung fibrosis, sarcoidosis, interstitial lung disease and pulmonary hypertension<br>• History of lung transplant or actively listed for transplant<br>• Pneumonia and/or moderate or severe COPD exacerbation 8 weeks prior to Visit 1[c]<br>• Use of maintenance ICS treatment within the past 12 months<br>• Participants with known hypersensitivity to LAMA, LABA or ICS or any component of MDI<br>• History of heart transplant or actively listed for transplant<br>• Implanted left ventricular assist device or implant anticipated in <3 months<br>• Unstable or life-threatening cardiac disease, including:<br>○ A MI or unstable angina in last 8 weeks<br>○ Unstable or life-threatening cardiac arrhythmia requiring intervention in past 8 weeks[c]<br>• End-stage renal disease requiring renal replacement therapy or<br>eGFR <20 mL/min/1.73 m^2<br>• History of lung cancer and/or treatment for lung cancer within the 5 years prior to Visit 1

	• Any life-threatening condition with a life expectancy <5 years<br>• Unable to abstain from protocol-defined prohibited medications<br>• Participation in another clinical study<br>• Currently pregnant or breastfeeding (females)
Study Type and Design	• Type: Interventional<br>• Allocation: Randomised<br>• Design: Double-blind, parallel group, multi-centre, multinational, event-drive<br>• Masking: Quadruple (participant, care provider, investigator, outcomes assessor)
Date of First Enrollment	21 February 2024
Sample Size	Planned enrollment of approximately 5000
Recruitment Status	Recruiting
Primary Outcome(s)	Time to first severe cardiac or COPD event across any of the following 3 groups of events: severe cardiac events (including HF acute healthcare visit/hospitalisation, MI hospitalisation), severe COPD exacerbation (i.e., requiring hospitalisation) or cardiopulmonary death (time frame: up to 3 years)

Key Secondary Outcomes	• Time to first severe COPD exacerbation event (time frame: up to 3 years)<br>• Time to first severe cardiac event (time frame: up to 3 years)<br>• Time to cardiopulmonary death (time frame: up to 3 years)<br>• Moderate/severe COPD exacerbation rate (time frame: over time, up to 3 years)<br>• Time to MI hospitalization or cardiac death (time frame: up to 3 years)<br>• Time to HF acute healthcare visit/hospitalization or cardiac death (time frame: up to 3 years)
Ethics Review	This study will be conducted in accordance with the consensus ethical principles derived from the Declaration of Helsinki (as amended at 64th WMA General Assembly, Fortaleza, Brazil, October 2013), Council for International Organisations of Medical Sciences International Ethical Guidelines, all applicable International Council for Harmonisation Good Clinical Practice guidelines and applicable laws and regulations. All relevant documents will be submitted to an institutional review board or independent ethics committee and approved before study initiation.
Completion date	3 March 2028 (estimated)
Summary Results	Summary results are not yet available as the study is ongoing. Relevant information will be posted to the appropriate clinical trial registries when available.
IPD sharing statement	• Plan to share: Yes; the Sponsor will accept requests, but this does not mean all requests will be granted.

	• Plan description: Qualified researchers can request access to anonymized individual patient-level data from AstraZeneca group of companies sponsored clinical trials via the request portal. All requests will be evaluated as per the AstraZeneca disclosure commitment: https://astrazenecagrouptrials.pharmacm.com/ST/Submission/Disclosure.<br>• Timeframe: AstraZeneca will meet or exceed data availability as per the commitments made to the European Federation of Pharmaceutical Industries and Associations Pharma Data Sharing Principles. For details of our timelines, please rerefer to our disclosure commitment at: https://astrazenecagrouptrials.pharmacm.com/ST/Submission/Disclosure.<br>• Access criteria: When a request has been approved AstraZeneca will provide access to the deidentified individual patient-level data in an approved sponsored tool. Signed Data Sharing Agreement (non-negotiable contract for data accessors) must be in place before accessing requested information. Additionally, all users will need to accept the terms and conditions to gain access. For additional details, please review the Disclosure Statements at https://astrazenecagrouptrials.pharmacm.com/ST/Submission/Disclosure.<br>• Supporting information types: study protocol and statistical analysis plan

[a]No more than 10% of participants should enter through only the HF criteria; NYHA Class II is defined as participants comfortable at rest; ordinary physical activity results in fatigue, palpitations, breathlessness or angina pectoris and Class III is defined as participants comfortable at rest, but with less than ordinary physical activity causing fatigue, dyspnoea, palpitations or angina.

[b]CV risk tools utilised in other countries/regions indicating equivalent risk (high on the scale) will also be accepted.

[c]Any participant who experiences unstable or life-threatening cardiac disease, pneumonia and/or moderate or severe COPD exacerbation during the run-in period will be excluded, but can be rescreened 8 weeks after the event's resolution.

ASCVD, atherosclerotic cardiovascular disease; BMI, body mass index; BP, blood pressure; BGF, budesonide/glycopyrrolate/formoterol fumarate; CAT, COPD Assessment test; COPD, chronic obstructive pulmonary disease; CV, cardiovascular; CVD, cardiovascular disease; ICS, inhaled corticosteroid; eGFR, estimated glomerular filtration rate; EU CT, European Union Clinical Trials; FEV_1, forced expiratory volume in 1 second; FVC, forced vital capacity; GFF, glycopyrronium/formoterol fumarate dihydrate; HF, heart failure; HDL-C, high-density lipoprotein cholesterol; ICS, inhaled corticosteroid; LABA, long-acting β_2-agonist; LAMA, long-acting muscarinic antagonist; LDL-C, low-density lipoprotein cholesterol; MDI, metered-dose inhaler; MI, myocardial infarction; NYHA, New York Heart Association; SCORE2, Systematic Coronary Risk Evaluation; SCORE2-OP, Systematic Coronary Risk Evaluation-Older Persons.
