# Supplementary material for: Effect of budesonide/glycopyrronium/formoterol fumarate dihydrate on cardiopulmonary outcomes in COPD: rationale and design of the THARROS trial
Source: ERJ Open Res. 2025 Dec 22;11(6):00324-2025. doi: 10.1183/23120541.00324-2025 (PMC12720155; doi:10.1183/23120541.00324-2025)
Supplement: Supplementary file 2 [file 00324-2025.SUPPLEMENT2.pdf]

Effect of budesonide/glycopyrronium/formoterol fumarate dihydrate on cardiopulmonary outcomes in COPD: rationale and design of the THARROS trial

Why are we doing this research?

People living with COPD are more likely to experience both heart- and lung-related medical problems (i.e., cardiopulmonary problems) compared with people who have not been diagnosed with COPD

A treatment known as **BGF** combines three medicines (**B**udesonide, **G**lycopyrronium and **F**ormoterol fumarate dihydrate) in a single inhaler

- BGF reduces the chance of COPD flare-ups (also known as exacerbations), improves lung function and reduces the risk of dying when compared with a treatment known as **GFF**, which combines two medicines (**G**lycopyrronium and **F**ormoterol **F**umarate dihydrate)

Large clinical studies have not tested how **BGF** versus **GFF** might improve combined heart- and lung-related measures in people living with COPD who are at high risk of experiencing cardiopulmonary problems

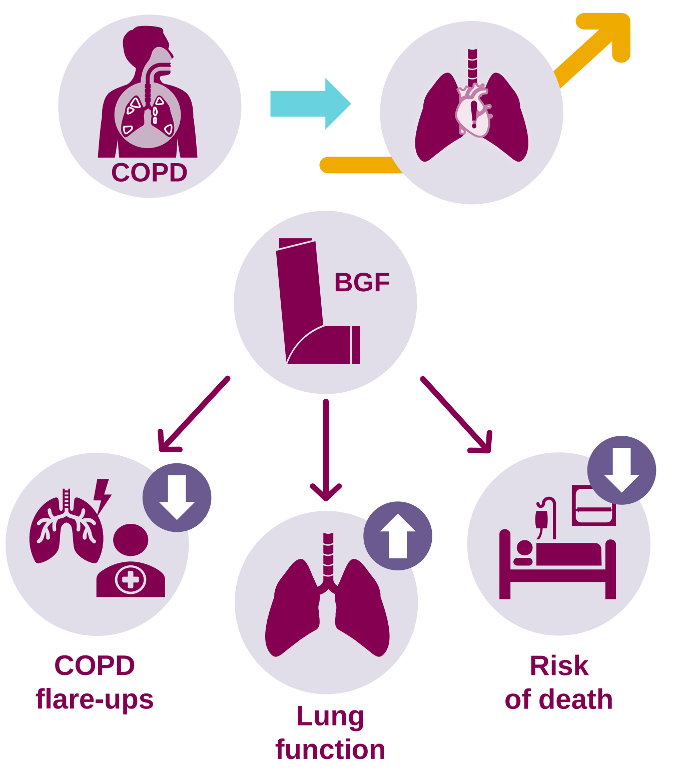

What will we measure?

The THARROS study will:

- Examine the benefits of **BGF** versus **GFF** with a novel measure based on a range of heart- and lung-related outcomes
- Use design features that: 1) focus on the most relevant patient population and 2) facilitate participation

How will we do this?

The THARROS study will be a large clinical study conducted in multiple countries

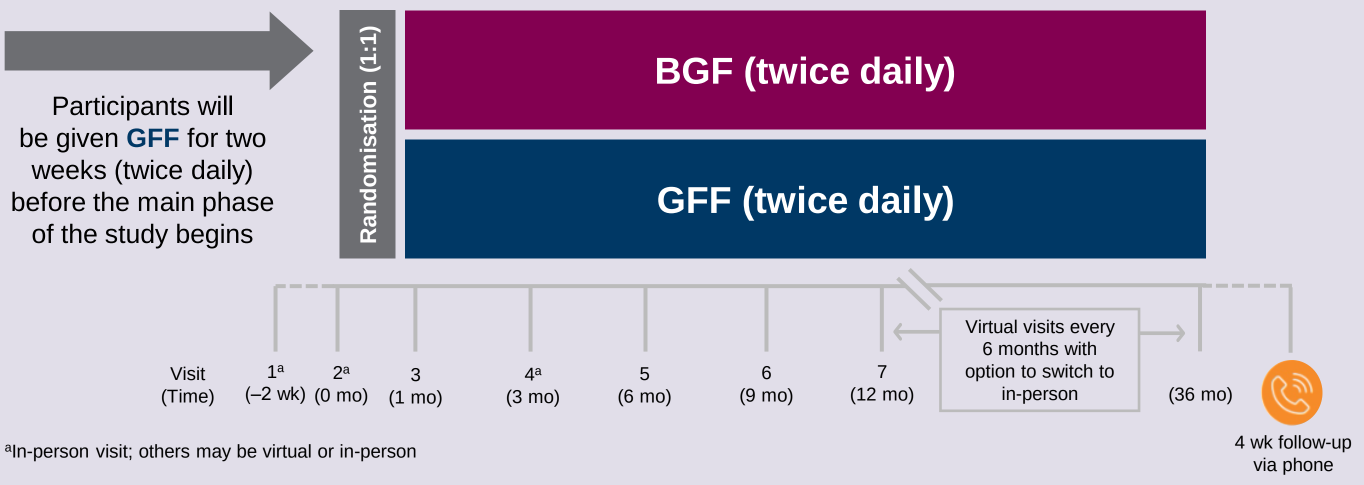

[a]In-person visit; others may be virtual or in-person

THARROS will give participants the option to have some study visits either in person or virtually to increase the convenience of participation

Key inclusion criteria

- 40–80 years old
- Lung-related criteria
  - Symptomatic COPD (CAT ≥10)
  - Current or former smoker
  - Blood eosinophils (i.e., white blood cells) >100 cells/mm³
- Heart-related criteria
  - Established heart disease (i.e., heart attack, coronary artery disease) or risk factors for heart disease (i.e., high blood pressure, diabetes)

There is no requirement for a history of COPD flare-ups

Key exclusion criteria

- Diagnosis of another lung disease (e.g., asthma)
- Inhaled corticosteroid (ICS) treatment in the last year
- Significant or advanced kidney failure
- Heart transplant or implanted device
- Transplant or cancer of lung
- Any life-threatening condition

Results to be measured

THARROS will use a new combined measure to capture both the heart- and lung-related benefits of **BGF** versus **GFF**

This will measure the time taken for the participant to have their first severe heart- or lung-related event, including a combination of the following outcomes:

Time to first healthcare visit or hospital stay caused by heart failure (when the heart is damaged and cannot pump as effectively as normal) or heart attack

Time to first severe COPD flare-up

Time to death cause by a heart- or lung-related medical problem

What makes this study unique?

- THARROS will specifically assess a group of participants with COPD who are already at risk of heart-related problems
- Using a combined measure of heart- and lung-related outcomes (that has not been used in previous COPD studies) will expand our knowledge of the benefits of **BGF** in people living with COPD
- Including participants who have **not** had flare-ups will help us to understand if participants with less severe COPD benefit from treatments that combine three medicines like **BGF**
- Excluding participants who have recently used ICS will help focus on patients considered less likely to be prescribed BGF
- Allowing virtual visits will encourage participation by making it more convenient to be involved in the study

Why will this be important?

The unique study design and measures used in THARROS will provide new information about the benefits of BGF for people living with COPD at high risk of COPD flare-ups and serious heart events like heart failure and heart attacks
